# Dietary fibre in relation to asthma, allergic rhinitis and sensitization from childhood up to adulthood

**DOI:** 10.1002/clt2.12188

**Published:** 2022-08-17

**Authors:** Emmanouela Sdona, Sandra Ekström, Niklas Andersson, Niclas Håkansson, Alicja Wolk, Marit Westman, Marianne van Hage, Inger Kull, Erik Melén, Anna Bergström

**Affiliations:** ^1^ Institute of Environmental Medicine Karolinska Institutet Stockholm Sweden; ^2^ Centre for Occupational and Environmental Medicine Stockholm Sweden; ^3^ Department of Surgical Sciences Uppsala University Uppsala Sweden; ^4^ Department of Medicine Solna Division of Immunology and Allergy Karolinska Institutet Stockholm Sweden; ^5^ Department of Clinical Immunology and Transfusion Medicine Karolinska University Hospital Stockholm Sweden; ^6^ Department of Clinical Science and Education Södersjukhuset Karolinska Institutet Stockholm Sweden; ^7^ Sachs' Children and Youth Hospital Södersjukhuset Stockholm Sweden

**Keywords:** allergic rhinitis, asthma, cohort, dietary fibre, sensitization

## Abstract

**Background:**

Dietary fibre may reduce the risk of allergy. Our aim was to investigate the association between fibre intake in childhood, asthma, allergic rhinitis and IgE sensitization up to adulthood.

**Methods:**

The individual fibre intake of 2285 participants from the Swedish population‐based birth cohort BAMSE was estimated between 98‐ and 107‐item food frequency questionnaires at ages 8 and 16 years, respectively. At 8, 16 and 24 years, asthma and allergic rhinitis symptoms were assessed by questionnaires, and sensitization to common allergens by serum IgE. Longitudinal associations were analysed by generalized estimating equations, adjusting for potential confounders.

**Results:**

An inverse overall association was indicated between fibre intake at 8 years and allergic rhinitis symptoms up to 24 years (OR per 5 g/d 0.86; 95% CI 0.77–0.96), particularly in combination with airborne (0.74; 0.62–0.89) and food (0.69; 0.54–0.88) allergen sensitization. Higher fibre intake was also associated with specific allergen sensitization, for example, birch (0.77; 0.67–0.88) and soy (0.68; 0.53–0.87). No association was observed with asthma. Regarding sources, fruit (0.79; 0.67–0.94) and other (potatoes, chips/popcorn, legumes, and nuts, 0.71; 0.50–0.99), but not cereal or vegetable fibre were associated with allergic rhinitis. In additional analyses, including long‐term fibre intake at 8 and 16 years, excluding participants with food‐related allergic symptoms to examine reverse causation, as well as adjusting for antioxidant intake, associations were attenuated and became non‐significant.

**Conclusion:**

Higher fibre intake in mid‐childhood may be inversely associated with allergic rhinitis and sensitization to specific allergens up to adulthood. However, avoidance of food triggers of allergic symptoms in allergic rhinitis patients may contribute to the protective associations.

## 1 INTRODUCTION

Epidemiological studies have shown that a high intake of dietary fibre is associated with a reduced risk of several chronic diseases, such as cardiovascular diseases, type 2 diabetes and cancer.^1^, ^2^, ^3^, ^4^ Fibre‐rich foods, including grains, fruits, vegetables and nuts, may also influence the development of allergic diseases, for example, by modulating the gut immunologic response, through the anti‐inflammatory effects of whole grains, and increased bio‐accessibility of antioxidants.^5^, ^6^, ^7^ In this respect, dietary fibre has been described to stimulate the growth of beneficial bacterial species and enhance the bacterial synthesis of immune‐modulating compounds, such as short chain fatty acids (SCFAs), that contribute to a healthy colonic microbiota ecosystem.^8^

However, a recent systematic review has shown that the epidemiological evidence for an association between fibre intake, asthma and allergic rhinitis is limited and inconclusive.^9^ Moreover, most of the results come from cross‐sectional studies in adult populations, while studies in children are scarce.^10^ In the aforementioned systematic review, two cross‐sectional studies on asthma among children were identified with inconsistent findings; one study from the US National Health and Nutrition Survey (NHANES) reporting inverse associations between fibre intake and asthma among children aged 2–11 years,^11^ and one Australian study reporting no association between fibre intake and self‐reported wheeze among adolescents.^12^ To our knowledge, there are no prospective studies on fibre intake in childhood or adolescence and subsequent development of allergic diseases.

Therefore, our aim was to investigate the association between dietary fibre intake in childhood and asthma, allergic rhinitis and IgE sensitization up to young adulthood, using repeated assessments in a prospective cohort study.

## 2 METHODS

### 2.1 Study design

The study was conducted within the prospective birth cohort BAMSE.^13^ In the BAMSE study, 4089 infants aged on average 2 months from predefined areas of Stockholm, Sweden were recruited (1994–1996). The children were subsequently followed with repeated questionnaires on lifestyle factors, selected exposures, and symptoms of allergic diseases up to age 24 years. At ages 4, 8, 16 and 24 years, participants were invited to clinical examinations, which included blood sampling using standardized methods. Sera have been analysed for specific IgE to common airborne (Phadiatop®) and food allergens (fx5®), using the ImmunoCAP System (Thermo Fisher Scientific, Uppsala, SE).^14^ In total, 3064 (75% of original cohort) young adults answered the questionnaire and 2234 (55%) provided blood samples at the 24‐year follow‐up. The study was approved by the Ethics Committee of Karolinska Institutet, Stockholm, Sweden (approval number 2016/1380‐31/2), and written informed consent was obtained.

### 2.2 Dietary fibre intake assessment

Diet at age 8 years was assessed using a food frequency questionnaire (FFQ) including 98 foods and beverages frequently consumed in Sweden, answered by a parent (57%), a parent together with the child (40%), or by another person (e.g., a grandparent). Participants were asked how often, on average, they had consumed each type of food or beverage during the past 12 months using 10 pre‐specified response categories ranging from “never” to “≥ three times/day” (FFQ in the Online Appendix).^15^ At age 16 years, the adolescents themselves answered a web‐based FFQ (TeenMeal‐Q) including 107 food items, which was designed for this age group.^16^ Similar FFQs as the ones used at 8 and 16 years have been validated in adults and have shown good validity with correlation coefficients for fibre intake of 0.71 and 0.65, respectively.^17^, ^18^, ^19^

For each FFQ, the frequency of consumption of the food items was converted into average daily consumption. The individual fibre intake was assessed by multiplying the frequency of consumption of each food item by its fibre content per serving, using composition values obtained from the Swedish Food Composition Database and summarized over foods and beverages.^20^ Total energy intake was summarized over the whole diet and calculated using food composition data from the Swedish Food Agency.^20^ Fibre intake was adjusted for energy intake using the residuals method and is presented per mean total energy intake (1900 kcal/day).^21^ Food items used in the calculation of fibre intake at 8 years are presented in Table S1 in the supplement.

### 2.3 Assessment of outcomes

Asthma and symptoms of allergic rhinitis (AR) were assessed based on questionnaires (parental reports at 8 years and self‐reports at 16 and 24 years). Participants were considered to have asthma if they had at least two of the following three criteria: ever doctor's diagnosis of asthma, at least one episode of wheeze and/or breathing difficulties, and/or asthma medication use in the last 12 months.^22^ AR symptoms were defined as symptoms from eyes or nose after exposure to furred pets or pollen (without having a flu) in the last 12 months.^22^ Sensitization to airborne allergens was defined as a positive Phadiatop®, and sensitization to food allergens as a positive fx5® result (IgE ≥ 0.35 kU_A_/l).^13^ Sera that scored positive for a mix were further analysed for the single allergens included in the mix (Phadiatop®: birch, timothy, mugwort, cat, dog, horse, house dust mite, mold; fx5®: milk, egg, soy, peanut, cod fish, wheat), as previously described.^13^ Sensitization to single allergens was defined as IgE ≥ 0.35 kU_A_/l. All samples were analysed at the Department of Clinical Immunology, Karolinska University Hospital Solna, Stockholm, Sweden.

To elucidate associations with asthma and symptoms of AR in combination with IgE sensitization, categorical variables were created. Participants were categorized as no asthma and no sensitization, no asthma but sensitization, no sensitization but asthma, asthma and sensitization, and corresponding categories were created for AR.

Prevalence refers to the total number of participants with the outcome at a specific follow‐up. Incidence refers to the number of participants with the outcome at a specific follow‐up, without fulfilling the definition of the outcome at the previous follow‐up.

### 2.4 Statistical analyses

Spearman rank correlation was used to describe the correlation between energy‐adjusted total and specific fibre intake at 8 years, as well as between energy‐adjusted total intakes at 8 and 16 years. Differences in selected characteristics of study participants were analyzed in relation to average fibre intake at 8 and 16 years by *chi* ^2^ and *t*‐test, as appropriate.

Associations between total fibre intake at 8 years, as well as fibre sources, and asthma, AR symptoms and IgE sensitization to airborne and food allergens up to 24 years were analyzed by generalized estimating equations (GEE) with a binomial family, a logit link function, and an unstructured correlation matrix. Prevalence and incidence of outcomes were examined in separate models. GEE models take the correlation between repeated measurements on the same individual into account. “Overall associations” combine a within‐subject relationship with a between‐subjects relationship, resulting in one single regression coefficient, while an interaction between time and exposure is used to estimate “age‐specific associations”.^23^ Analyses were stratified by sex and tested for interaction. Associations were presented per 5 g/day increments. For comparison, an ordinary sized apple or orange contains 3–3.5 g of fibre. Linear associations were tested by the Wald test. Associations for which there was evidence of non‐linearity were further flexibly modelled using restricted cubic splines with three knots.

Associations between fibre intake, asthma and symptoms of AR in combination with IgE sensitization were analyzed using multinomial GEE.

To use the repeated exposure assessment, associations with long‐term fibre intake as an updated lagged exposure were analyzed by GEE, that is, fibre at 8 years was modelled against outcomes at 8 and 16 years and fibre at 16 years was modelled against outcomes at 24 years^24^

We identified potential confounding factors from previous literature and then refined our selection by using a directed acyclic graph (DAG) approach (Figure S1).^25^, ^26^ In the multivariable model, we adjusted for sex, total energy intake (kcal/day) at 8 years, parental education (elementary school, high school, university), parental ethnicity (born in or outside of Scandinavia), parental history of atopic disease (yes/no) and maternal smoking in pregnancy and/or infancy (yes/no). The covariates described in the supplement were tested but did not affect the estimates. Fibre sources were mutually adjusted by inclusion in the same model.

Associations were further tested after exclusion of participants with reported allergic symptoms related to fruits or vegetables, and/or avoidance of any of these due to allergic symptoms, to investigate potential disease‐related modification of exposure,^27^ and after adjustment for dietary total antioxidant capacity (TAC, described in the supplement), to investigate confounding by antioxidants.^16^

Participants with baseline questionnaire data, data on fibre intake at ages 8 and 16 years with a mean energy intake within ±3 log SD and completed questionnaires at 8, 16 and/or 24 years were included in the study population (*n* = 2285); participants with available specific IgE to common allergens at 8, 16 and/or 24 years were included in sensitization analyses (*n* = 2026). The flow chart of the study is shown in Figure S2. For statistical analyses, STATA V.16 (StataCorp TX, USA) and R V.4.1.2 (R Core Team, 2021) were used.

## 3 RESULTS

The study population (*n* = 2285) was generally comparable to the original cohort (*n* = 4089) with regards to distribution of selected characteristics (Table S2).

The median energy‐adjusted total fibre intake at age 8 years was 18.0 g/day (females 18.3 vs. males 17.6, *p* < 0.001), and at 16 years 17.1 g/day (females 18.2 vs. males 16.3, *p* < 0.001). The Spearman correlation coefficients between total energy‐adjusted fibre and cereal, fruit, vegetable and other (including potatoes, chips/popcorn, legumes, and nuts) fibre at 8 years were 0.44, 0.64, 0.53 and 0.19, respectively. The correlation between total intakes at 8 and 16 years was 0.21 (*p* < 0.001). Cereal, fruit, vegetable and other sources contributed 40%, 24%, 12% and 16% of the total at 8 years, and 43%, 11%, 13% and 21% of the total at 16 years, respectively.

Participants with high fibre intake (≥median) at ages 8 and 16 years were more likely females and had higher socioeconomic status (in terms of living area at birth, parental work and education, maternal age, smoking during pregnancy and/or infancy) compared to those with low intake (Table 1). Additionally, they had a lower energy intake but were more likely overweight or obese at 8 years, had a high level of physical activity and were less likely to smoke at 16 years (all *p* < 0.05).

**TABLE 1:** Distribution of demographic and lifestyle characteristics of study participants (*n* = 2285) by average fibre intake at ages 8 and 16 years

	Average fibre intake at 8 and 16 years		
	<17.7 g/d	≥17.7 g/d	
	*N* = 1142	*N* = 1143	
	*n* (%)	*n* (%)	*p* value^a^
Male sex	664 (58.1)	480 (42.0)	<0.001
Living area at birth^b^			
Urban	327 (28.7)	396 (34.9)	
Suburban	811 (71.3)	738 (65.1)	0.002
Maternal age <26 years	93 (8.1)	64 (5.6)	0.016
Parental allergic disease	372 (32.7)	339 (30.0)	0.163
Parent white collar worker	948 (83.8)	983 (87.1)	0.029
Parent with university education	572 (50.1)	682 (59.7)	<0.001
Parent born out of Scandinavia	159 (14.0)	192 (16.9)	0.053
Maternal smoking in pregnancy and/or infancy	167 (14.6)	119 (10.4)	0.002
Breastfeeding ≥4 m	894 (79.7)	924 (82.3)	0.116
Older siblings	553 (48.4)	519 (45.4)	0.148
Day nursery at age 2 years	818 (72.8)	845 (75.0)	0.248
8 years			
Overweight or obesity	199 (17.4)	258 (22.6)	0.002
Physical activity >2 times/w	185 (16.2)	162 (14.2)	0.180
Allergy to fruits and vegetables^c^	115 (10.9)	111 (10.6)	0.825
Use of multivitamins	476 (42.2)	482 (42.8)	0.798
Energy intake, Kcal/d, mean (SD)	1927 (460)	1889 (462)	0.049
16 years			
Overweight or obesity	181 (17.7)	155 (14.9)	0.081
High physical activity^d^	716 (66.7)	762 (69.8)	0.003
Allergy to fruits and vegetables^c^	140 (15.7)	173 (19.1)	0.053
Smoking	143 (12.5)	110 (9.6)	0.027
Energy intake, Kcal/d, mean (SD)	1934 (777)	1905 (755)	0.370
24 years			
Overweight or obesity	196 (24.8)	171 (21.4)	0.101
High physical activity^d^	445 (56.7)	480 (58.4)	0.196
Smoking	214 (21.5)	186 (18.7)	0.115

^a^ *p‐values* were calculated with the *chi* ^2^ test for categorical variables and *t*‐test for continuous variables.

^b^ Urban: central parts of Stockholm (Norrmalm); suburban: northwestern parts of Stockholm (municipalities Järfälla, Solna or Sundbyberg).

^c^ Allergic symptoms related to fruits and/or vegetables, or avoidance of any fruit or vegetable due to allergic symptoms.

^d^ Levels of physical activity according to IPAQ guidelines: High: ≥7 h/week of moderate to vigorous activity or ≥3.5 h/week of vigorous activity.

The prevalence of asthma increased between the ages of 8 (10.8%) and 16 years (14.8%), and the rate was not much different at age 24 years (14.6%). There was also a rise in the prevalence of AR, and this trend continued throughout the study (14.2% at 8, 25.5% at 16, 30.9% at 24 years). The prevalence of airborne allergen sensitization increased from 8 (26.2%) to 16 years (44.6%) and remained relatively stable at 24 years (44.9%), while food allergen sensitization prevalence decreased (20.3% at 8, 13.7% at 16, 10.4% at 24 years; Table S3). Prevalence of single allergen sensitization is shown in Table S4. Among participants with asthma at ages 8, 16 and 24 years, 60.9%, 72.5% and 76.3% were sensitized to airborne, and 43.6%, 28.5% and 27.2% to food allergens, while among those with AR symptoms at respective ages, 86.0%, 89.0% and 81.0% were sensitized to airborne, and 45.8%, 27.4% and 18.5% to food allergens, respectively.

### 3.1 Associations between fibre intake at 8 years and asthma, AR symptoms and IgE sensitization up to 24 years

A higher fibre intake at 8 years was associated with reduced odds of prevalent AR symptoms up to 24 years (OR per 5 g/d 0.86; 95% CI 0.77–0.96; Table 2). In contrast, no association was observed with asthma. Regarding fibre sources, higher fruit and other fibre intakes were inversely associated with AR symptoms up to 24 years (OR 0.79; 95% CI 0.67–0.94 and OR 0.71; 95% CI 0.50–0.99, respectively), while no association was observed with cereal or vegetable fibre intakes (Table 2). No interaction between fibre intake and gender regarding the association with the outcomes was observed (data not shown).

**TABLE 2:** Overall associations between fibre intake at age 8 years, asthma, allergic rhinitis symptoms and IgE sensitization from 8 up to 24 years

	Fibre intake at 8 years OR per 5 g/day (95% CI)	
	Prevalence	Incidence
Asthma		
Total fibre	0.94 (0.82–1.08)	0.97 (0.82–1.15)
Cereal fibre	1.02 (0.82–1.26)	1.24 (0.95–1.62)
Fruit fibre	0.85 (0.68–1.05)	0.86 (0.66–1.12)
Vegetable fibre	1.03 (0.72–1.47)	0.83 (0.52–1.30)
Other fibre	1.20 (0.80–1.79)	1.40 (0.85–2.30)
Allergic rhinitis symptoms		
Total fibre	0.86 (0.77–0.96)	0.98 (0.86–1.11)
Cereal fibre	0.93 (0.77–1.11)	0.90 (0.73–1.11)
Fruit fibre	0.79 (0.67–0.94)	0.98 (0.81–1.17)
Vegetable fibre	1.03 (0.77–1.38)	1.09 (0.79–1.51)
Other fibre	0.71 (0.50–0.99)	0.80 (0.54–1.18)
Sensitization to airborne allergens		
Total fibre	0.93 (0.83–1.04)	0.91 (0.79–1.06)
Cereal fibre	0.89 (0.74–1.07)	0.82 (0.64–1.04)
Fruit fibre	0.93 (0.79–1.10)	0.98 (0.78–1.23)
Vegetable fibre	1.00 (0.75–1.35)	0.99 (0.67–1.46)
Other fibre	0.93 (0.65–1.31)	0.87 (0.54–1.41)
Sensitization to food allergens		
Total fibre	0.91 (0.79–1.04)	0.83 (0.63–1.09)
Cereal fibre	1.08 (0.87–1.35)	0.87 (0.56–1.36)
Fruit fibre	0.89 (0.72–1.09)	0.81 (0.53–1.24)
Vegetable fibre	0.72 (0.50–1.05)	0.94 (0.46–1.90)
Other fibre	0.96 (0.63–1.47)	0.43 (0.17–1.07)

*Note*: Generalized estimating equations (GEE) models adjusted for sex, total energy intake, parental education, ethnicity, history of atopic disease, and smoking in pregnancy and/or infancy. OR (95% CI): odds ratio (95% confidence interval).

In age‐specific analyses, we observed inverse associations between a higher total fibre intake at 8 years and AR symptoms at 8 years (OR 0.76; 95% CI 0.65–0.89), a higher fruit fibre intake and AR symptoms at 8 (OR 0.66; 95% CI 0.50–0.86) and 16 years (OR 0.77; 95% CI 0.64–0.94), and the observed ORs moved towards unity with increasing age. In contrast, the association between other fibre intake and AR symptoms was significant at 24 years (OR 0.64; 95% CI 0.43–0.97; Table S5).

In an analysis of single allergens, a higher fibre intake at 8 years was inversely associated with sensitisation to a number of allergens, including birch, mugwort, horse, soy, and peanut up to the age of 24 years (Table 3). For birch and soy, there were also comparable associations at 16 and 24 years (Table S6).

**TABLE 3:** Overall associations between fibre intake at age 8 years and IgE sensitization to specific airborne and food allergens from 8 up to 24 years

	Fibre intake at 8 years
	OR per 5 g/day (95% CI)
Sensitization to airborne allergens	
Birch	0.77 (0.67–0.88)
Timothy	0.96 (0.84–1.09)
Mugwort	0.78 (0.66–0.93)
Cat	0.92 (0.79–1.06)
Dog	0.90 (0.78–1.03)
Horse	0.77 (0.64–0.93)
Mite	1.05 (0.90–1.24)
Mold	0.76 (0.52–1.11)
Sensitization to food allergens	
Milk	1.01 (0.84–1.21)
Egg	0.93 (0.74–1.17)
Soy	0.68 (0.53–0.87)
Peanut	0.74 (0.60–0.91)
Fish	1.43 (0.71–2.89)
Wheat	0.80 (0.65–1.00)

*Note*: Generalized estimating equations (GEE) models adjusted for sex, total energy intake, parental education, ethnicity, history of atopic disease, and smoking in pregnancy and/or infancy. OR (95% CI): odds ratio (95% confidence interval).

Evidence for non‐linearity (*p* < 0.05) was observed for the association between fibre intake, AR symptoms and IgE sensitization, and the shape of the associations was explored using restricted cubic splines (Figure S3). Upon visual inspection of the splines, we did not observe considerable deviation from linearity, and therefore associations were assumed to be linear.

### 3.2 Associations between fibre intake at 8 years, asthma and AR symptoms in combination with IgE sensitization up to 24 years

**FIGURE 1:**
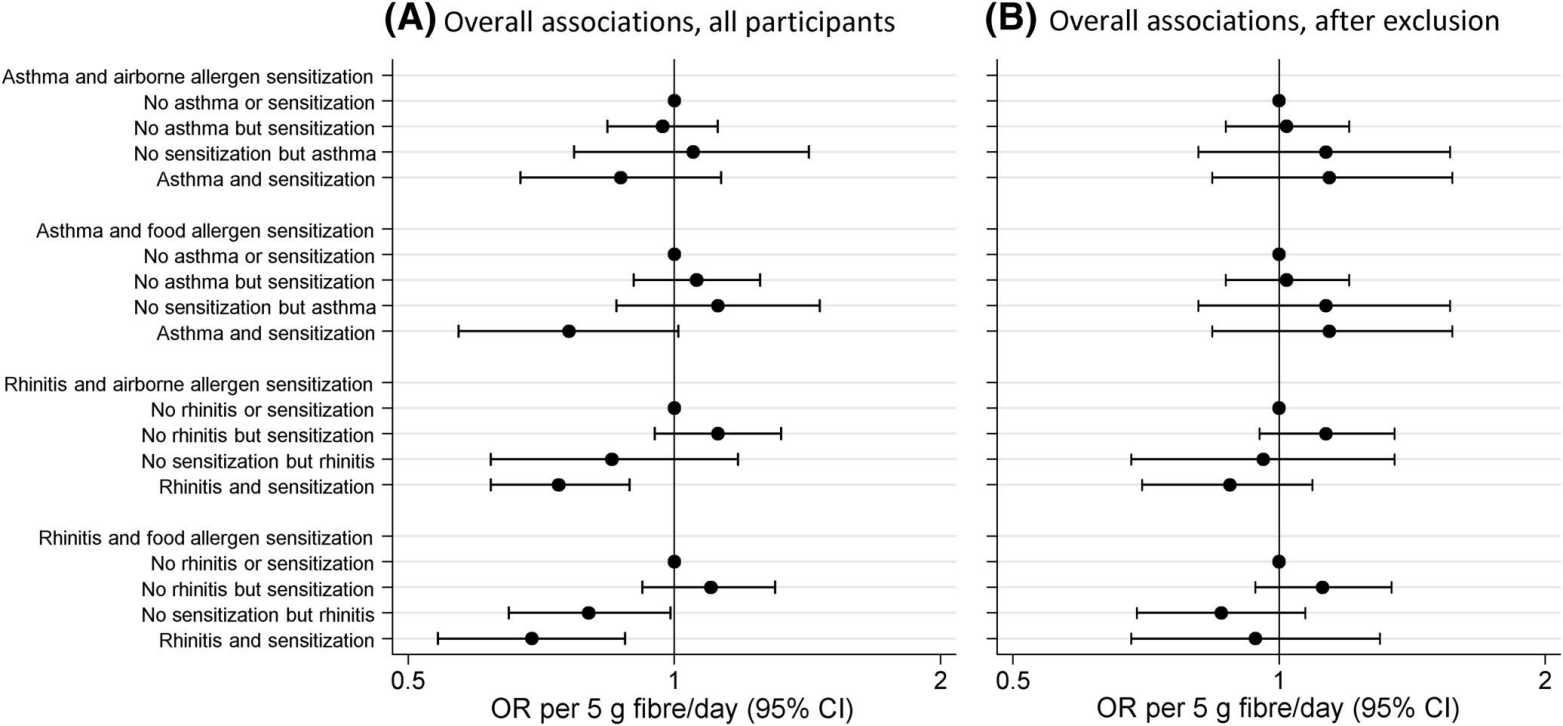
Overall associations between total fibre intake at 8 years, asthma and allergic rhinitis symptoms in combination with IgE sensitization from 8 up to 24 years, (A) all participants, (B) after exclusion of participants with food‐related allergic symptoms. Multinomial generalized estimating equations (GEE) analyses adjusted for sex, total energy intake, parental education, ethnicity, history of atopic disease, and smoking in pregnancy and/or infancy. OR (95% CI): odds ratio (95% confidence interval)

An inverse association with total fibre intake at 8 years was observed among participants with AR who were sensitized to airborne (OR 0.74; 95% CI 0.62–0.89), and food allergens (OR 0.69; 95% CI 0.54–0.88) up to 24 years (Figure 1A).

### 3.3 Associations between long‐term fibre intake and asthma, AR symptoms and IgE sensitization up to 24 years

The updated lagged exposure model showed non‐significant overall tendencies consistent with the 8‐year fibre model (Table 4). In single allergen sensitization analyses, a similar pattern to the 8‐year model was observed, but with no significant associations at age 24 years (Table S7).

**TABLE 4:** Longitudinal overall associations between long‐term fibre at 8 and 16 years, asthma, allergic rhinitis symptoms and IgE sensitization from 8 up to 24 years

	Long‐term fibre intake at 8 and 16 years
	OR per 5 g/day (95% CI)
Asthma	
Prevalence	0.99 (0.92–1.08)
Incidence	0.90 (0.77–1.04)
Allergic rhinitis symptoms	
Prevalence	0.96 (0.90–1.03)
Incidence	0.99 (0.89–1.10)
Sensitization to airborne allergens	
Prevalence	0.95 (0.90–1.00)
Incidence	0.90 (0.78–1.03)
Sensitization to food allergens	
Prevalence	0.95 (0.87–1.04)
Incidence	0.94 (0.74–1.20)

*Note*: Generalized estimating equations (GEE) models adjusted for sex, total energy intake, parental education, ethnicity, history of atopic disease, and smoking in pregnancy and/or infancy. Total fibre intake at 8 years was modelled against outcomes at 8 and 16 years, and total fibre intake at 16 years was modelled against outcomes at 24 years, while total energy intake was handled similarly. OR (95% CI): odds ratio (95% confidence interval).

### 3.4 Sensitivity analyses

After exclusion of participants with allergy to fruits or vegetables, and/or avoidance of any of these due to allergic symptoms at 8 years (*n* = 226), the associations moved towards the null and became non‐significant (Table S8, Figure 1B).

Birch pollen‐sensitized children frequently develop hypersensitivity reactions to specific food items, which may lead to avoidance of these items.^28^ In our study, after exclusion of birch pollen‐sensitized children at 8 years (*n* = 315, 16% of participants) who reported food‐related allergic symptoms (*n* = 111), associations disappeared (data not shown).

Nevertheless, when we tested the associations with single allergens included in Phadiatop® and fx5® after exclusion of participants with food‐related allergic symptoms, the association between total fibre intake at 8 years, birch (OR 0.86; 95% CI 0.74–1.00) and soy (OR 0.73; 95% CI 0.54–0.99) allergen sensitization up to 24 years remained (Table S9).

Adjustment of analyses with dietary TAC at 8 years attenuated the associations, which became non‐significant, for example, total fibre intake at 8 years and AR symptoms up to 24 years OR 0.92; 95% CI 0.79–1.08.

## 4 DISCUSSION

In our study of 2285 young adults from the population‐based birth cohort BAMSE, higher dietary fibre intake at 8 years was associated with reduced odds of AR up to 24 years. Additionally, a higher total fibre intake was associated with reduced odds of sensitization to specific airborne and food allergens, for example, birch and soy. No consistent association with asthma was observed. After exclusion of participants with food‐related allergic symptoms, associations became non‐significant, indicating that disease‐related modification of consumption may have contributed to the results; the inverse association with birch and soy allergen sensitization though remained.

To the best of our knowledge, our study is the first to examine the association between fibre intake, asthma, AR and IgE sensitization from childhood up to adulthood. Moreover, we examined disease‐related modification of exposure, which constitutes an important methodological challenge in studies of diet and allergy.^15^ Children may avoid foods or eat them less often if they induce immediate symptoms, which might result in an apparent protective association of these foods with allergic disease. Pollen‐food allergy syndrome, caused by IgE directed at cross‐reacting allergens found in pollens and plant foods, such as fruits, vegetables and nuts, often coexists with AR symptoms.^29^ The pattern of cross‐reactivity differs between countries.^30^ In Sweden, the most common cross‐reactivity is found between birch tree pollen and apple, stone fruit, and carrot.^31^ In our study, 35% of birch pollen‐sensitized children reported food‐related allergic symptoms, and by excluding these, the protective association between fibre intake and AR symptoms disappeared. Although disease‐related modification of exposure is likely to be a greater problem in cross‐sectional studies, it may also be of importance in prospective studies. In our study, the fact that an association was observed with prevalent, but not incident, AR may further indicate this.

Epidemiological studies have reported inverse associations between fibre intake and asthma among adults. In the US NHANES population, a low fibre diet was associated with increased odds of wheeze,^32^ and in the Korean NHANES population a higher fibre intake was associated with reduced odds of asthma.^33^ Fewer asthma symptoms and greater asthma control among adults with higher dietary fibre intake were observed in cross‐sectional analyses of a large French cohort.^34^ Epidemiological evidence on the protective role of fibre on allergic asthma development is reinforced by animal studies, as well as clinical studies of fibre supplementation.^35^ In a mouse model, a high‐fibre diet led to a marked suppression of allergic airways disease, which was also transmitted to the offspring generation.^36^ Additionally, epidemiological studies on diets rich in fruits and vegetables have reported inverse associations with asthma.^37^ However, these studies have not focused on fibre per se, and thus the beneficial effect might have been attributed to other constituents of the diet with anti‐inflammatory or antioxidant properties. In our study, we did not observe a significant association between fibre intake and asthma, while adjustment by dietary TAC indicated that antioxidants, or a better diet quality in general, might have contributed to the results.

Although few studies have examined dietary fibre in relation to AR or IgE sensitization, a high adherence to the Mediterranean diet has been associated with protection against AR among children in a cross‐sectional study from Greece.^38^ In adults, a cross‐sectional study in the Korean NHANES population reported lower prevalence of AR symptoms, such as watery rhinorrhoea, and dog allergen sensitization among males with higher fibre intake, but not among females,^33^ while a cross‐sectional study of women from the Osaka Maternal and Child Health Study reported no association between fibre intake and AR.^39^ Very few publications describe fibre sources as compared with total fibre intake, in which stronger associations between grains and some health outcomes have been described.^40^, ^41^ In our study, total, fruit, and other fibre intake were inversely associated with AR symptoms, while no association was observed with cereal or vegetable fibre intake. Gender‐specific associations between fibre intake and health outcomes have also been reported.^42^ In our study, total fibre intake was higher among females, but no effect modification by gender was observed. Associations were attenuated when we evaluated long‐term fibre intake instead of fibre intake at 8 years, which could be attributed to the different exposure window or the different FFQ design at 16 years.

An association between fibre intake, AR, and IgE sensitization to specific allergens is biologically plausible. High dietary fibre has been associated with lower concentration of systemic inflammation markers, such as CRP and IL‐6.^43^, ^44^ A plant‐based, high fibre diet has been shown to promote the growth of beneficial bacterial species in the gut, leading to increased local and systemic levels of SCFAs, which can influence inflammatory responses and epigenetically modify DNA.^5^, ^45^ Upper and lower airways share common pathological mechanisms, and a high fibre intake in a murine model attenuated allergic symptoms of nasal rubbing and sneezing.^46^ Further research is needed on the interplay between airway and gut microbiota, and the mechanisms of potentially differential effects of dietary fibre on specific allergens.^47^, ^48^

Major strengths of our study include the population‐based prospective longitudinal design, long follow‐up time with limited loss to follow‐up. Repeated data on exposure and outcomes made it possible to study prevalence and incidence of outcomes and account for disease‐related modification of exposure, while the longitudinal analysis made it possible to study both overall, taking into account the correlation between repeated assessments on the same individual into account, and age‐specific associations. Additionally, we were able to explore the association with IgE sensitization to specific airborne and food allergens. The individual fibre intake was calculated using information from validated FFQs and our results are in line with other Swedish studies.^49^, ^50^ The recommended daily intake of dietary fibre is 10–40 g for children and adolescents, depending on age, sex and energy intake.^40^, ^51^ In Sweden, the recommended intake is 2–3 g/MJ, with an increasing intake from school age up to adolescence.^52^ Although absolute intake may not be accurately estimated by FFQs, and different FFQ designs were used at 8 and 16 years, it is still possible to rank the participants according to their intake, while repeated dietary assessment may better capture long‐term intake and reduce exposure misclassification.^53^ We were also able to consider many confounders, using a DAG approach. Despite this, residual confounding cannot be completely ruled out.

Regarding limitations, selection bias should not be an important problem, as there were small differences in the distribution of characteristics of the study population compared to the total cohort. Although asthma and symptoms of AR were assessed through questionnaires, which could lead to some misclassification, probably non‐differential with regards to fibre intake, well‐established definitions (MeDALL) were used. In addition, most children in the study also provided a blood sample for IgE analyses at age 4 years and parents were informed about the results. Thus, the possibility that this knowledge influenced subsequent food choices cannot be excluded.

In conclusion, in this prospective cohort we observed that a higher fibre intake in mid‐childhood was associated with reduced odds of AR and sensitization to specific airborne and food allergens up to adulthood, although avoidance of food triggers of allergic symptoms in allergic rhinitis patients may contribute to the protective associations. Therefore, further studies on childhood dietary fibre intake in relation to subsequent allergic disease are needed, taking disease‐related exposure modification into account. Furthermore, since the prevalence of pollen‐food allergy syndrome is expected to rise,^28^ increased awareness is required among clinicians and patients, so that excluded foods are replaced with tolerated ones, and thus the recommended dietary fibre intake can be attained.

## AUTHOR CONTRIBUTIONS

**Emmanouela Sdona**: Conceptualization (Equal); Formal analysis (Lead); Methodology (Lead); Project administration (Lead); Visualization (Lead); Writing – original draft (Lead). **Sandra Ekstrom**: Validation (Equal); Writing – review & editing (Equal). **Niklas Andersson**: Software (Equal); Visualization (Equal); Writing – review & editing (Equal). **Niclas Hakansson**: Software (Equal); Writing – review & editing (Equal). **Alicja Wolk**: Writing – review & editing (Equal); **Marit Westman**: Writing – review & editing (Equal). **Marianne van Hage**: Formal analysis (Equal); Writing – review & editing (Equal). **Inger Kull**: Data curation (Equal); Writing – review & editing (Equal). **Erik Melen**: Data curation (Equal); Writing – review & editing (Equal). **Anna Bergstrom**: Conceptualization (Equal); Data curation (Equal); Funding acquisition (Lead); Investigation (Lead); Resources (Lead); Supervision (Lead). Writing – review & editing (Equal).

## CONFLICT OF INTEREST

The authors have no conflicts of interest to disclose.

## Supporting information

Supplementary Material 1

Supplementary Material 2
