# Supplementary material for: Dietary fibre in relation to asthma, allergic rhinitis and sensitization from childhood up to adulthood
Source: Clin Transl Allergy. 2022 Aug 17;12(8):e12188. doi: 10.1002/clt2.12188 (PMC9382355; doi:10.1002/clt2.12188)
Supplement: Supplementary file 1 — Supplementary Material 1 [file CLT2-12-e12188-s001.docx]

**Supplementary Material**

**Dietary fibre in relation to asthma, allergic rhinitis and sensitization from childhood up to adulthood**

Sdona E^1^, Ekström S^1,2^, Andersson N^1^, Håkansson N^1^, Wolk A^1,3^, Westman M^4^, van Hage M^4,5^, Kull I^6,7^, Melén E^1,6,7^, Bergström A^1,2^

^1^ Institute of Environmental Medicine, Karolinska Institutet, Stockholm, Sweden

^2^ Centre for Occupational and Environmental Medicine, Region Stockholm, Stockholm, Sweden

^3^ Department of Surgical Sciences, Uppsala University, Uppsala, Sweden

^4^ Department of Medicine Solna, Division of Immunology and Allergy, Karolinska Institutet, Stockholm, Sweden

^5^ Department of Clinical Immunology and Transfusion Medicine, Karolinska University Hospital, Stockholm, Sweden

^6^ Department of Clinical Science and Education, Södersjukhuset, Karolinska Institutet, Stockholm, Sweden

^7^ Sachs’ Children and Youth Hospital, Södersjukhuset, Stockholm, Sweden

Corresponding author: Emmanouela Sdona, Unit of Environmental Epidemiology, Institute of Environmental Medicine, Karolinska Institutet, 171 77 Stockholm, Sweden. Phone number: +46 702 417 034. E-mail: [emmanouela.sdona@ki.se](mailto:emmanouela.sdona@ki.se)

*Description of covariates*

Living area at birth: Urban areas included central parts of Stockholm (Norrmalm). Suburban areas included northwestern parts of Stockholm (municipalities Järfälla, Solna and Sundbyberg).

Allergic heredity: Doctor diagnosed asthma and/or hay fever in combination with reported allergy to pollen or pets in one or both parents (at baseline).

Parental occupation: Socioeconomic status of the household (at baseline) based on parental occupation, categorized as professional worker (white collar worker) or non-professional worker (blue collar worker), according to Statistics Sweden “Socioeconomic division (SEI); Reports on Statistical Coordination 1982:4”.

Parental education: Highest education level of the household (at baseline), based on maternal and paternal education level, in two categories (elementary school or high school, university).

Parental origin outside of Scandinavia: One or both parents born outside of Sweden, Norway, Denmark, or Finland.

Maternal smoking during pregnancy and/or infancy: The mother smoked at least one cigarette per day at any point of time during pregnancy and/or in infancy.

Maternal BMI in early pregnancy: Maternal body mass index (kg/m^2^) recorded at the first visit to the antenatal-care clinic around week 10 in pregnancy.

Older siblings: Any older siblings in the household/family.

Length of gestation: Full term (>=39 weeks).

Mode of delivery: Vaginal or caesarean delivery.

Breastfeeding ≥ 4 months: Exclusive breastfeeding for at least four months.

Childcare: Day nursery at age 2 years.

Fruit and vegetable intake: Total fruit and vegetable intake in servings per day at ages 8 or 16 years.

Fish intake: Total fish intake in servings per day at ages 8 or 16 years.

Multivitamin use: Multivitamin supplement use at ages 8 or 16 years.

Smoking: Daily or occasional smoking at ages 16 or 24 years.

Physical activity: At age 8 years, participation in physical activity or sports three or more times per week as reported by the parent. At ages 16 and 24 years, self-reported amount of vigorous (e.g. lifting heavy weights, aerobics, or high-speed bicycling) and moderate (e.g. bicycling at normal speed, carrying light objects) physical activity in the last 12 months (mean of summer and winter season). Levels of physical activity was defined according to IPAQ guidelines.^1^ High levels of physical activity: ≥ 7 hours/week of moderate to vigorous activity or ≥ 3.5 hours/week of vigorous activity. Moderate: ≥ 2.5 hours/week of moderate to vigorous activity. Low: < 2.5 hours/week of moderate to vigorous activity.

Overweight: Weight and height was measured at clinical investigations at ages 8, 16 and 24 years. At 8 and 16 years, overweight was defined per gender- and age-specific cut-off values for body mass index proposed by the International Obesity Task Force.^2^ At 24 years, overweight was defined as body mass index ≥ 25 kg/m^2^.

Food allergy: Allergic symptoms related to fruits or vegetables, and/or avoidance of any of these due to allergic symptoms, as reported at ages 8 or 16 years.

Total antioxidant capacity (TAC): Individual dietary TAC at age 8 years, estimated by combining the information on the frequency of consumption of specific food items with the information from a database of common foods analysed with the oxygen radical absorbance capacity (ORAC) method^3^ on the average ORAC content [μmol Trolox equivalents (TE)/day] of age-specific portion sizes, and energy-adjusted using the residuals method.^4^ Overall, in the 98-item FFQ, there were 35 food items (including all fruits and vegetables) with available ORAC values.^5^

**Table S1.** Food items from the 8-year food frequency questionnaire included in specific fibre sources

| **Specific fibre** | **Respective food items** |
| --- | --- |
| Fruit fibre | oranges/citrus fruits, apples/pears, bananas, other fruits, berries, juice, jam/marmalade, stewed fruit/fruit soup |
| Vegetable fibre | white/red cabbage, cauliflower, broccoli/brussels sprouts, lettuce, tomatoes, cucumbers, spinach/kale, onion/leek, green peas, rutabaga/beetroot, carrots |
| Cereal fibre | crispbread, white bread, wholemeal bread, bread with flaxseed, bread loaf, oatmeal, other porridge or gruel, breakfast cereals, spaghetti/macaroni/noodle, pancakes, pizza, rice, buns, Danish pastry, biscuit, snap, cake |
| Other fibre | pea soup/puree, brown/white beans/soya/lentils, boiled potatoes, mashed potatoes, fried potatoes, potatoes baked in the oven, French fries, chips/popcorn, nuts/almonds, pea nuts |

**Figure S1.** Directed acyclic graph to study covariates and potential structural confounding bias for the association between total fibre intake and incident asthma

**
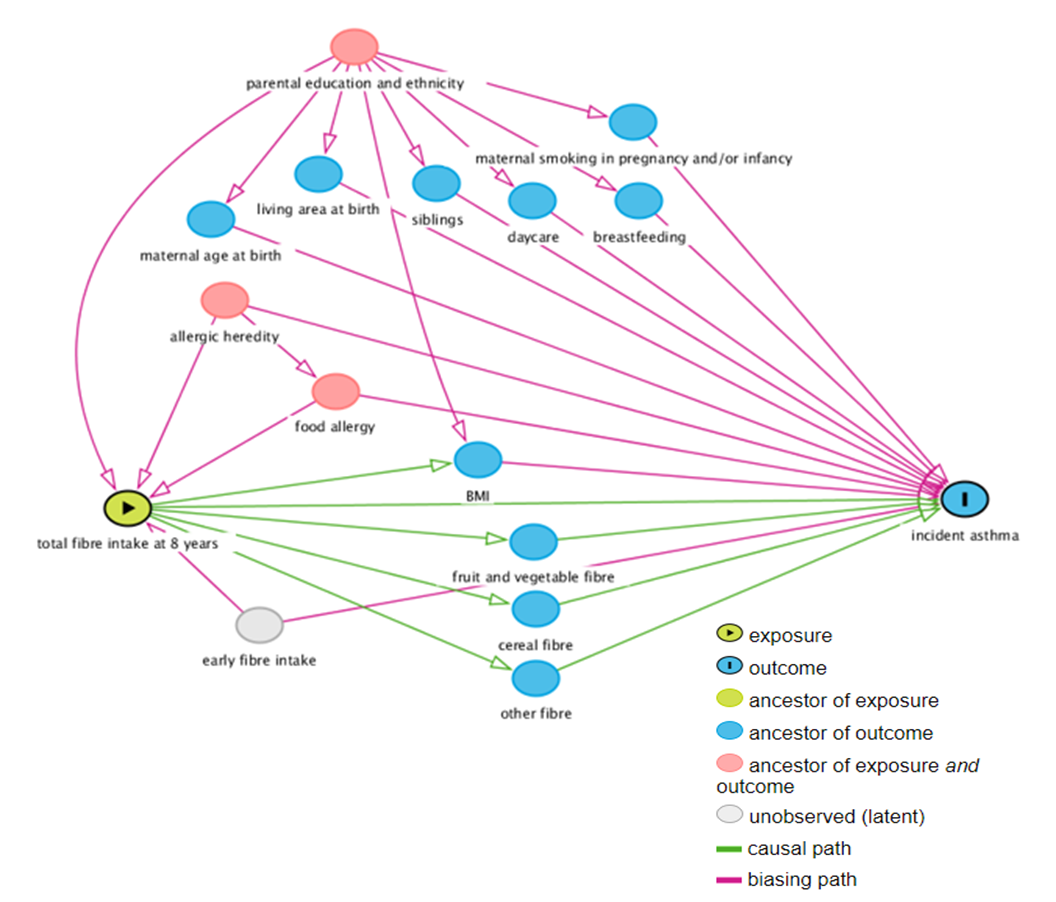
**

**Figure S2.** Flow chart of the study


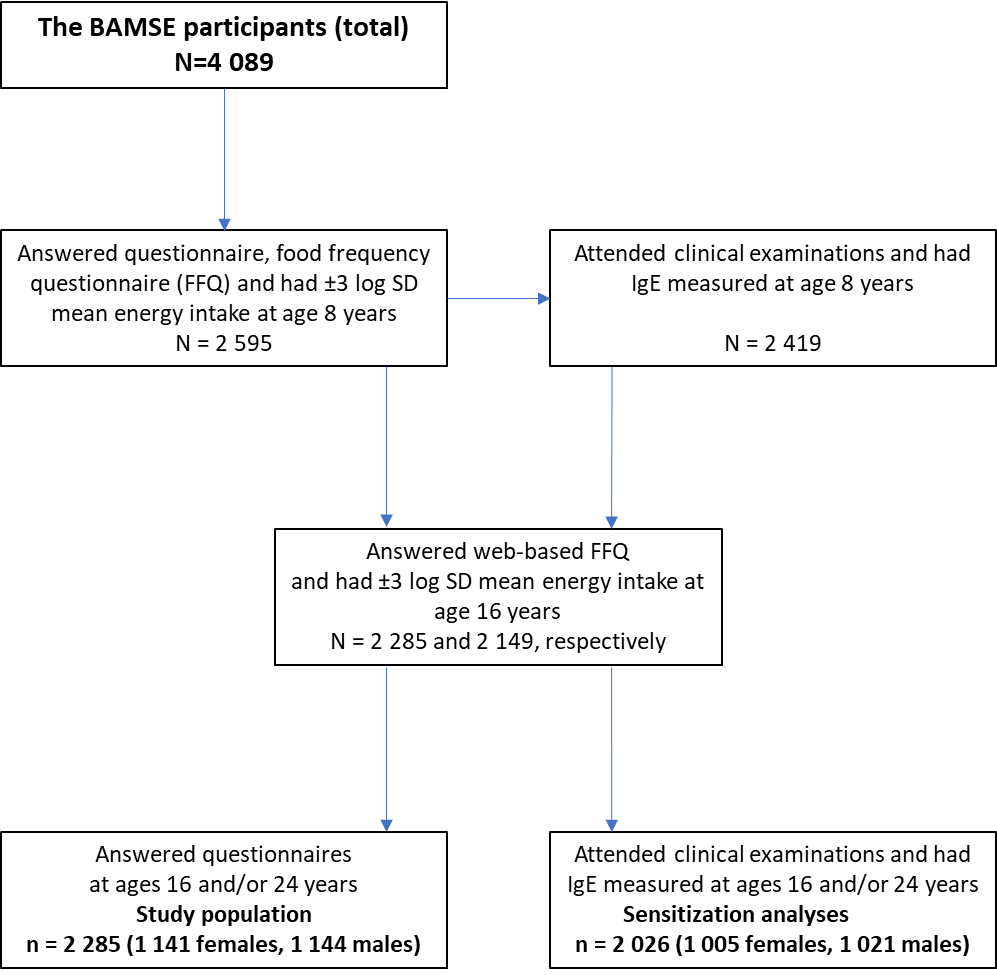


Due to the longitudinal design of the study, data on the outcome were required from at least one time-point (16 or 24 years). Participants with missing data at one time-point were included in analyses of overall associations, but not in the respective age-specific association. At age 24 years, participants with complete questionnaires were 1 994 (1 040 females and 954 males) and participants with measured IgE were 1 491 (794 females and 697 males). The higher proportion of females at age 24 years is due to higher loss to follow-up rate among males.

**Table S2.** Distribution of selected characteristics in the original cohort and in the study population

|  | **Total cohort** | | **Study population** | | **Sensitization analyses** | |
| --- | --- | --- | --- | --- | --- | --- |
|  | **n=4 089** | | **n=2 285** | | **n=2 026** | |
| **Selected characteristics** | **n** | **%** | **n** | **%** | **n** | **%** |
| Male sex (n=4 089) | 2 065 | 50.5 | 1 144 | 50.1 | 1 021 | 50.4 |
| Living area at birth^†^ (n=4 071): - Urban | 1 205 | 29.6 | 723 | 31.8 | 642 | 31.9 |
| - Suburban | 2 866 | 70.4 | 1 549 | 68.2 | 1 372 | 68.1 |
| Parent with university education (n=4 082) | 2 161 | 52.9 | 1 254 | 54.9 | 1 137 | 56.2 |
| Parent born out of Scandinavia (n=3 398) | 543 | 16.0 | 351 | 15.5 | 321 | 15.9 |
| Parent with allergic disease (n=4 041) | 1 200 | 29.7 | 711 | 31.4 | 649 | 32.3 |
| Caesarean section^‡^ (n=4 011) | 507 | 12.6 | 274 | 12.2 | 248 | 12.5 |
| Exclusive breastfeeding ≥ 4 months (n=3 919) | 3 116 | 79.5 | 1 818 | 81.0 | 1 618 | 81.3 |
| Older siblings (n=4 088) | 1 980 | 48.4 | 1 072 | 46.9 | 948 | 46.8 |
| Maternal smoking in pregnancy and/or infancy (n=4 086) | 563 | 13.8 | 286 | 12.5 | 239 | 11.8 |
|  | **mean** | **SD** | **mean** | **SD** | **mean** | **SD** |
| Maternal age, y (n=4 088) | 30.7 | 4.5 | 31.0 | 4.5 | 31.0 | 4.5 |
| Maternal BMI^‡^, kg/m^2^ (n=3 533) | 22.9 | 3.3 | 22.9 | 3.2 | 22.8 | 3.1 |
| Birth weight^‡^, g (n=3 998) | 3 535 | 552 | 3 539 | 550 | 3 542 | 549 |
| Gestational age^‡^, w (n=4 005) | 39.5 | 1.8 | 39.5 | 1.8 | 39.5 | 1.8 |

^†^ Urban: central parts of Stockholm (Norrmalm); suburban: northwestern parts of Stockholm County (the municipalities Järfälla, Solna or Sundbyberg).  ^‡^ Variables obtained from the medical birth register. 95% CI: 95% confidence interval, SD: standard deviation.

**Table S3.** Descriptive information on asthma, allergic rhinitis symptoms and IgE sensitization from age 8 up to 24 years

|  | **8 years** | | | **16 years** | | | | **24 years** | | | |  |
| --- | --- | --- | --- | --- | --- | --- | --- | --- | --- | --- | --- | --- |
|  | **Females** | **Males** | **Total** | | **Females** | **Males** | **Total** | | **Females** | **Males** | **Total** | |
|  | **N=1 141** | **N=1 144** | **N=2 285** | | **N=1 141** | **N=1 144** | **N=2 285** | | **N=1 040** | **N=954** | **N=1 994** | |
| **Asthma, n (%)** | | | | | | | | | | | | |
| Prevalence | 103 (9.0) | 144 (12.6) | 247 (10.8) | | 178 (15.6) | 160 (14.0) | 338 (14.8) | | 166 (15.6) | 126 (13.2) | 292 (14.6) | |
| Incidence | NA | NA | NA | | 111 (9.7) | 72 (6.3) | 183 (8.0) | | 56 (5.4) | 46 (4.8) | 102 (5.1) | |
| **Allergic rhinitis symptoms, n (%)** | | | | | | | | | | | | |
| Prevalence | 146 (12.8) | 179 (15.7) | 325 (14.2) | | 262 (23.0) | 320 (28.0) | 582 (25.5) | | 330 (31.7) | 287 (30.1) | 617 (30.9) | |
| Incidence | NA | NA | NA | | 156 (13.7) | 175 (15.3) | 331 (14.5) | | 134 (12.9) | 90 (9.4) | 224 (11.2) | |
|  |  |  |  | |  |  |  | |  |  |  | |
|  | **N=1 005** | **N=1 021** | **N=2 026** | | **N=961** | **N=962** | **N=1 923** | | **N=794** | **N=697** | **N=1 491** | |
| **Sensitization to airborne allergens, n (%)** | | | | | | | | | | | |  |
| Prevalence | 226 (22.5) | 305 (29.9) | 531 (26.2) | | 377 (39.2) | 481 (50.0) | 858 (44.6) | | 304 (38.3) | 365 (52.4) | 669 (44.9) | |
| Incidence | NA | NA | NA | | 171 (17.8) | 204 (21.2) | 375 (19.5) | | 30 (3.8) | 29 (4.2) | 59 (4.0) | |
| **Sensitization to food allergens, n (%)** | | | | | | | | | | | | |
| Prevalence | 218 (21.7) | 193 (18.9) | 411 (20.3) | | 127 (13.2) | 137 (14.2) | 264 (13.7) | | 71 (8.9) | 84 (12.1) | 155 (10.4) | |
| Incidence | NA | NA | NA | | 31 (3.2) | 43 (4.5) | 74 (3.9) | | 16 (2.0) | 18 (2.6) | 34 (2.3) | |

Prevalent cases: the total number of cases at the respective age. Incident cases: cases with the outcome at the respective age, without fulfilling the definition of the outcome at the previous follow-up. Percentages were calculated in relation to the total number of subjects who contributed to the analyses at the respective follow-up. NA: not applicable (due to non-availability of exposure data on fibre intake before 8 years).

**Table S4.** Descriptive information on prevalence of IgE sensitization to common airborne and food allergens from age 8 up to 24 years

|  | **8 years** | **16 years** | **24 years** |
| --- | --- | --- | --- |
|  | **Total** | **Total** | **Total** |
|  | **N=2 026** | **N=1 923** | **N=1 491** |
| **Sensitization to airborne allergens, n (%)** | | | |
| Birch | 315 (15.6) | 462 (24.0) | 391 (26.2) |
| Timothy | 290 (14.3) | 528 (27.5) | 440 (29.5) |
| Mugwort | 150 (7.4) | 255 (13.3) | 168 (11.3) |
| Cat | 261 (12.9) | 358 (18.6) | 316 (21.2) |
| Dog | 239 (11.8) | 427 (22.2) | 272 (18.2) |
| Horse | 144 (7.1) | 199 (10.4) | 158 (10.6) |
| Mite: - *Dermatophagoides pteronyssinus* | 90 (4.4) | 248 (12.9) | 180 (12.1) |
| *- Dermatophagoides farinae* |  | 228 (11.9) | 174 (11.7) |
| Mold | 46 (2.3) | 37 (1.9) | 18 (1.2) |
| **Sensitization to food allergens, n (%)** | | | |
| Milk | 207 (10.2) | 66 (3.4) | 33 (2.2) |
| Egg | 123 (6.1) | 51 (2.7) | 34 (2.3) |
| Soy | 101 (5.0) | 103 (5.4) | 78 (5.2) |
| Peanut | 166 (8.2) | 157 (8.2) | 110 (7.4) |
| Fish | 9 (0.4) | 5 (0.3) | 5 (0.3) |
| Wheat | 122 (6.0) | 98 (5.1) | 65 (4.4) |

Sensitization defined as specific IgE ≥ 0.35 kUA/l.

**Table S5.** Age-specific associations between fibre intake at 8 years and allergic rhinitis symptoms from 8 up to 24 years

|  | **Fibre intake at 8 years** | | |
| --- | --- | --- | --- |
|  | **OR per 5 g/day (95% CI)** | | |
|  | **8 years** | **16 years** | **24 years** |
| Total fibre | 0.76 (0.65-0.89) | 0.87 (0.77-1.00) | 0.90 (0.79-1.02) |
| Cereal fibre | 0.96 (0.73-1.25) | 0.91 (0.74-1.14) | 0.92 (0.74-1.14) |
| Fruit fibre | 0.66 (0.50-0.86) | 0.77 (0.64-0.94) | 0.86 (0.71-1.06) |
| Vegetable fibre | 0.95 (0.63-1.42) | 1.08 (0.77-1.52) | 1.03 (0.72-1.48) |
| Other fibre | 0.71 (0.41-1.21) | 0.79 (0.53-1.19) | 0.64 (0.43-0.97) |

Generalized estimating equations (GEE) models adjusted for sex, total energy intake, parental education, ethnicity, history of atopic disease, and smoking in pregnancy and/or infancy.

OR (95% CI): odds ratio (95% confidence interval).

**Table S6.** Age-specific associations between fibre intake at 8 years and IgE sensitization to airborne and food allergens from 8 up to 24 years

|  | **Fibre intake at 8 years**  **OR per 5 g/day (95% CI)** | | | | |
| --- | --- | --- | --- | --- | --- |
|  | **8 years** | **16 years** | **24 years** | | |
| **Sensitization to airborne allergens** | | | |  |  |
| Birch | 0.71 (0.60-0.84) | 0.79 (0.69-0.91) | 0.78 (0.67-0.90) | | |
| Timothy | 0.89 (0.75-1.05) | 0.96 (0.84-1.11) | 0.99 (0.86-1.14) | | |
| Mugwort | 0.66 (0.52-0.84) | 0.84 (0.69-1.01) | 0.78 (0.62-0.98) | | |
| Cat | 0.94 (0.78-1.12) | 0.93 (0.80-1.09) | 0.90 (0.76-1.05) | | |
| Dog | 0.82 (0.68-1.00) | 0.89 (0.77-1.03) | 0.97 (0.81-1.15) | | |
| Horse | 0.82 (0.64-1.04) | 0.72 (0.58-0.88) | 0.84 (0.66-1.05) | | |
| Mite | 1.03 (0.75-1.41) | 1.03 (0.85-1.25) | 1.09 (0.90-1.33) | | |
| Mold | 0.80 (0.52-1.23) | 0.70 (0.41-1.20) | 0.88 (0.44-1.78) | | |
| **Sensitization to food allergens** | | | | |  |
| Milk | 1.04 (0.86-1.26) | 0.82 (0.55-1.23) | 1.03 (0.64-1.65) | | |
| Egg | 0.94 (0.75-1.17) | 0.90 (0.59-1.36) | 0.96 (0.59-1.55) | | |
| Soy | 0.66 (0.49-0.87) | 0.70 (0.52-0.92) | 0.68 (0.48-0.98) | | |
| Peanut | 0.68 (0.54-0.85) | 0.82 (0.66-1.01) | 0.70 (0.53-0.93) | | |
| Fish | 1.48 (0.83-2.64) | 1.01 (0.34-2.96) | 1.58 (0.60-4.19) | | |
| Wheat | 0.74 (0.57-0.96) | 0.99 (0.76-1.30) | 0.70 (0.47-1.05) | | |

Sensitization defined as specific IgE ≥ 0.35 kUA/l.

Generalized estimating equations (GEE) models adjusted for sex, total energy intake, parental education, ethnicity, history of atopic disease, and smoking in pregnancy and/or infancy.

OR (95% CI): odds ratio (95% confidence interval).

**Figure S3.** Overall associations between total fibre intake at 8 years and A. allergic rhinitis symptoms, B. IgE sensitization to specific allergens from 8 up to 24 years, flexibly modelled using restricted cubic splines with three knots (at percentiles 10, 50, 90, reference p10).

1. **Allergic rhinitis symptoms from 8 up to 24 years**

**
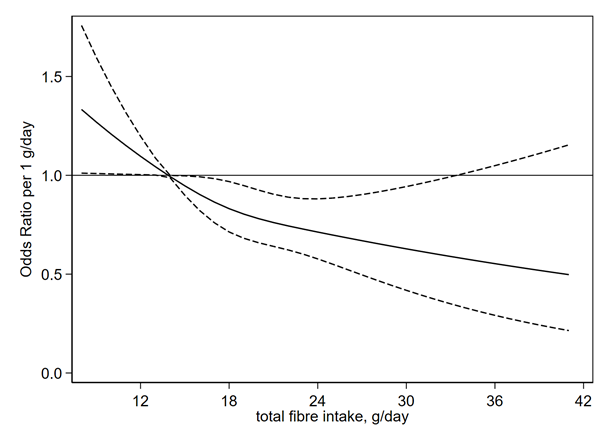
**

Odds ratios (OR) and 95% confidence intervals (95% CI) were estimated using GEE analyses adjusted for sex, total energy intake, parental education, ethnicity, history of atopic disease, and smoking in pregnancy and/or infancy. The solid line represents OR and the dashed lines represent 95% CI.

1. **IgE sensitization to specific allergens from 8 up to 24 years**


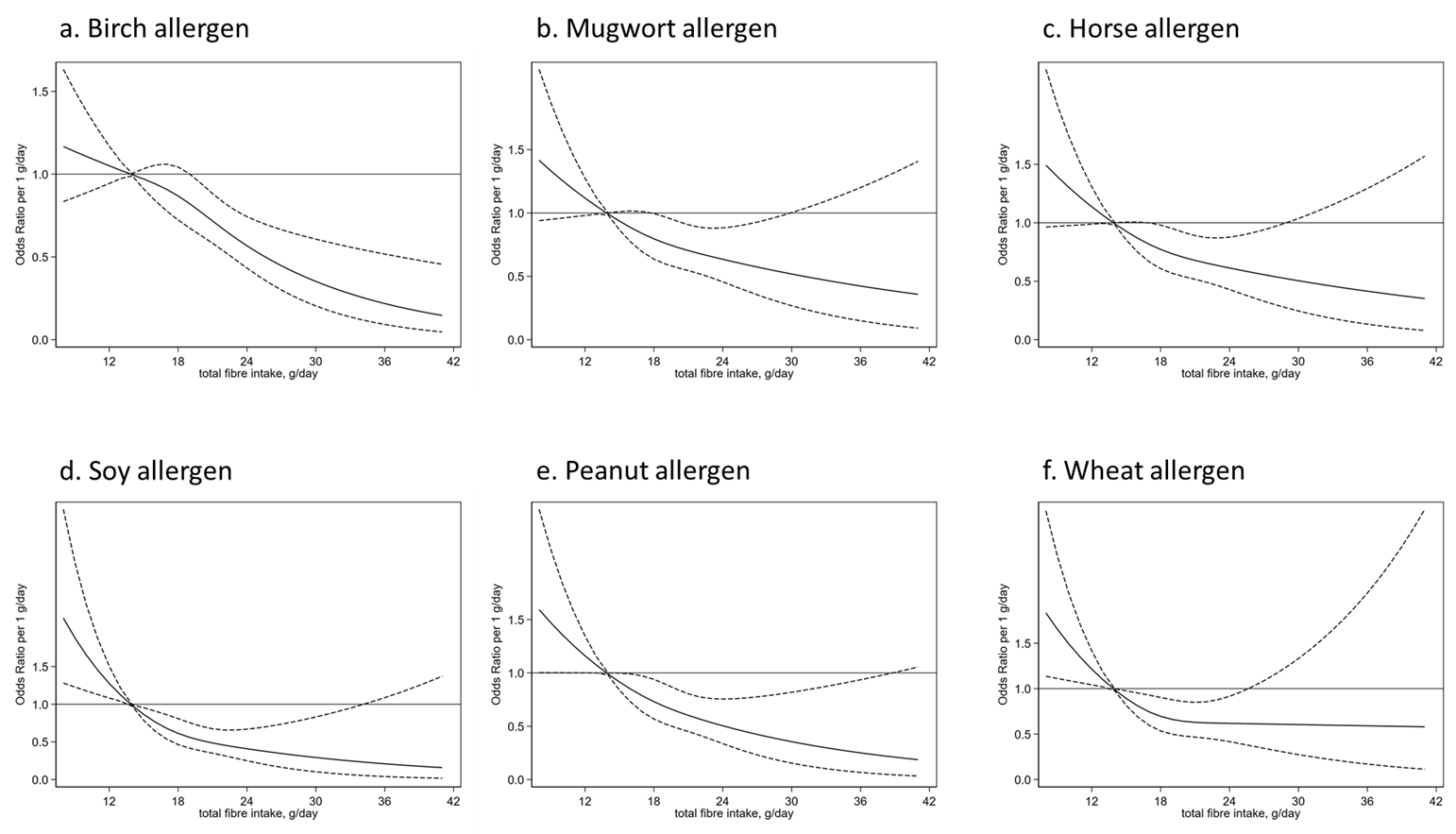


Odds ratios (OR) and 95% confidence intervals (95% CI) were estimated using GEE analyses adjusted for sex, total energy intake, parental education, ethnicity, history of atopic disease, and smoking in pregnancy and/or infancy. The solid line represents OR and the dashed lines represent 95% CI.

**Table S7.** Overall and age-specific associations between long-term fibre intake at 8 and 16 years and IgE sensitization to airborne and food allergens from 8 up to 24 years

|  | **Fibre intake at 8 and 16 years**  **OR per 5 g/day (95% CI)** | | | |  |
| --- | --- | --- | --- | --- | --- |
|  | **Overall** | **8 years** | **16 years** | **24 years** | |
| **Sensitization to airborne allergens** | | |  |  | |
| Birch | 0.96 (0.91-1.01) | 0.85 (0.76-0.96) | 0.95 (0.87-1.04) | 0.97 (0.91-1.05) | |
| Timothy | 1.00 (0.95-1.06) | 0.90 (0.78-1.03) | 0.98 (0.90-1.06) | 1.02 (0.95-1.09) | |
| Mugwort | 1.02 (0.92-1.12) | 0.76 (0.62-0.94) | 0.96 (0.83-1.11) | 1.10 (0.97-1.24) | |
| Cat | 0.99 (0.94-1.05) | 1.01 (0.87-1.16) | 1.01 (0.91-1.12) | 0.98 (0.92-1.05) | |
| Dog | 0.96 (0.90-1.04) | 0.84 (0.72-0.99) | 0.92 (0.83-1.02) | 1.01 (0.93-1.09) | |
| Horse | 0.94 (0.86-1.03) | 0.91 (0.74-1.12) | 0.82 (0.71-0.95) | 1.01 (0.92-1.11) | |
| Mite | 0.99 (0.89-1.10) | 1.00 (0.73-1.36) | 0.98 (0.83-1.16) | 0.99 (0.87-1.13) | |
| Mold | 0.87 (0.68-1.12) | 0.84 (0.62-1.15) | 0.75 (0.51-1.11) | 1.00 (0.75-1.33) | |
| **Sensitization to food allergens** | | |  |  | |
| Milk | 1.00 (0.86-1.17) | 1.04 (0.86-1.26) | 0.81 (0.56-1.19) | 1.04 (0.75-1.44) | |
| Egg | 0.99 (0.84-1.18) | 0.95 (0.77-1.17) | 0.94 (0.66-1.34) | 1.07 (0.82-1.39) | |
| Soy | 0.87 (0.75-1.01) | 0.78 (0.60-1.00) | 0.84 (0.67-1.06) | 0.93 (0.75-1.15) | |
| Peanut | 0.91 (0.81-1.01) | 0.81 (0.68-0.97) | 1.00 (0.84-1.18) | 0.90 (0.77-1.06) | |
| Fish | 1.67 (1.02-2.74) | 1.51 (0.82-2.79) | 1.02 (0.30-3.44) | 2.07 (0.97-4.39) | |
| Wheat | 0.92 (0.78-1.07) | 0.79 (0.62-1.00) | 1.12 (0.89-1.42) | 0.90 (0.69-1.17) | |

Generalized estimating equations (GEE) models adjusted for sex, total energy intake, parental education, ethnicity, history of atopic disease, and smoking in pregnancy and/or infancy.

Total fibre intake at 8 years was modelled against outcomes at 8 and 16 years, and total fibre intake at 16 years was modelled against outcomes at 24 years, while total energy intake was handled similarly.

OR (95% CI): odds ratio (95% confidence interval).

**Table S8.** Overall associations between fibre intake at 8 years and asthma, allergic rhinitis symptoms and IgE sensitization from 8 up to 24 years, after exclusion of participants with food-related allergic symptoms (n=226)

|  | **Fibre intake at 8 years** |
| --- | --- |
|  | **OR per 5 g/day (95% CI)** |
| **Asthma** | |
| Total fibre | 1.06 (0.92-1.23) |
| Cereal fibre | 1.06 (0.84-1.35) |
| Fruit fibre | 0.95 (0.75-1.19) |
| Vegetable fibre | 1.26 (0.86-1.85) |
| Other fibre | 1.34 (0.86-2.07) |
| **Allergic rhinitis symptoms** | |
| Total fibre | 0.96 (0.85-1.08) |
| Cereal fibre | 0.94 (0.78-1.14) |
| Fruit fibre | 0.94 (0.79-1.13) |
| Vegetable fibre | 1.05 (0.77-1.43) |
| Other fibre | 0.83 (0.57-1.20) |
| **Sensitization to airborne allergens** | |
| Total fibre | 1.00 (0.89-1.13) |
| Cereal fibre | 0.88 (0.72-1.06) |
| Fruit fibre | 1.05 (0.88-1.25) |
| Vegetable fibre | 1.09 (0.80-1.49) |
| Other fibre | 1.02 (0.71-1.49) |
| **Sensitization to food allergens** | |
| Total fibre | 1.01 (0.87-1.17) |
| Cereal fibre | 1.11 (0.87-1.40) |
| Fruit fibre | 1.02 (0.82-1.27) |
| Vegetable fibre | 0.75 (0.50-1.11) |
| Other fibre | 1.23 (0.79-1.93) |

Generalized estimating equations (GEE) models adjusted for sex, total energy intake, parental education, ethnicity, history of atopic disease, and smoking in pregnancy and/or infancy.

OR (95% CI): odds ratio (95% confidence interval).

**Table S9.** Overall associations between fibre intake at age 8 years and IgE sensitization to airborne and food allergens up to 24 years, after exclusion of participants with food-related allergic symptoms (n=226)

|  | **Fibre intake at 8 years**  **OR per 5 g/day (95% CI)** |
| --- | --- |
| **Sensitization to airborne allergens** | |
| Birch | 0.86 (0.74-1.00) |
| Timothy | 1.00 (0.87-1.15) |
| Mugwort | 0.87 (0.71-1.06) |
| Cat | 1.07 (0.91-1.25) |
| Dog | 1.04 (0.89-1.21) |
| Horse | 0.90 (0.73-1.12) |
| Mite | 1.12 (0.94-1.33) |
| Mold | 0.79 (0.50-1.27) |
| **Sensitization to food allergens** | |
| Milk | 1.04 (0.86-1.26) |
| Egg | 1.05 (0.82-1.35) |
| Soy | 0.73 (0.54-0.99) |
| Peanut | 0.86 (0.68-1.10) |
| Fish | 1.85 (0.84-4.09) |
| Wheat | 0.95 (0.74-1.22) |

Generalized estimating equations (GEE) models adjusted for sex, total energy intake, parental education, ethnicity, history of atopic disease, and smoking in pregnancy and/or infancy.

OR (95% CI): odds ratio (95% confidence interval).

**Reference List**

1. Guidelines for data processing and analysis of the International Physical Activity Questionnaire (IPAQ) - Short and long forms; 2005. <http://www.ipaq.ki.se>. Accessed 10 January 2022.

2. Cole TJ and Lobstein T. Extended international (IOTF) body mass index cut-offs for thinness, overweight and obesity. *Pediatr Obes*. 2012;7(4): 284-289.

3. Wu X, Beecher G, Holden J, et al. Lipophilic and hydrophilic antioxidant capacities of common foods in the United States. *J Agric Food Chem*. 2004;52:4026-4037.

4. Willett W, Howe G, Kushi L. Adjustment for total energy intake in epidemiologic studies. *Am J Clin Nutr*. 1997;65(4 Suppl):1220S-S1228.

5. Sdona E, Ekström S, Andersson N, et al. Fruit, vegetable and dietary antioxidant intake in school age, respiratory health up to young adulthood. *Clin Exp Allergy*. 2022;52(1):104-114.
