# Supplementary material for: Dietary fibre in relation to asthma, allergic rhinitis and sensitization from childhood up to adulthood
Source: Clin Transl Allergy. 2022 Aug 17;12(8):e12188. doi: 10.1002/clt2.12188 (PMC9382355; doi:10.1002/clt2.12188)
Supplement: Supplementary file 2 — Supplementary Material 2 [file CLT2-12-e12188-s002.pdf]

Barn Allergi Miljö Stockholm Epidemiologi PROJEKTET

Questions about the child's diet

Questionnaire for

Bamse identification number

Date when answering the FFQ |_2_|_0_|_0_|____| - |____|____| - |____|____|

f8dat

Who filled out the FFQ?

f8q_a

1 ☐ Mother
2 ☐ Father
3 ☐ The child
4 ☐ Other person, who? **f8q_b**
5 = mother + child
6 = father + child
7 = mother + father
8 = mother + father + child
9 = other person + child

Instructions

- Fill out the questionnaire together with your child.
- The questions cover what the child hav eaten at home and in school and includes snacks.

Du underlättar för oss om du skriver tydligt och markerar kryssrutorna klart.

Dietary habits during the last 12 months

f8q1 What type of milk do your child usually drink or eat, and how much per day or per week?

(1 glass = 2 dl)

			Glass per day	Glass per week
_1	☐	Milk ≤0.5% fat	a_1	b_1
_2	☐	Milk 1.5% fat	a_2	b_2
_3	☐	Milk 3% fat	a_3	b_3
_4	☐	Sour milk or yogurt	a_4	b_4
_5	☐	Low fat sour milk or yogurt	a_5	b_5
_6	☐	Chocolate milk (oboy)	a_6	b_6
_7	☐	Other? f8q1_7t	a_7	b_7

_8 ☐ Drink or eat milk, sour milk or yogurt **less** than once a week

_9 ☐ **Never** drink or eat milk, sour milk or yogurt

f8q2 What type of bread do your child usually eat, and how many slices per day or per week?

			Slices per day	Slices per week
_1	☐	Crispbread	a_1	b_1
_2	☐	White bread	a_2	b_2
_3	☐	Wholemeal bread	a_3	b_3
_4	☐	Bread with flaxseed	a_4	b_4
_5	☐	”Limpa” (type of bread)	a_5	b_5

f8q3 How many slices of bread with butter or margarine does your child eat per day or per week?

a ☐ slices per day

b ☐ slices per week

f8q4 How much butter/margarine does your child usually spread on his/her bread?

1 ☐ Thick

2 ☐ Fairly thick

3 ☐ Thin

4 ☐ Very thin

5 ☐ None at all

---

f8q5 What types fat does your child usually use on sandwiches?

(More than one option possible)

_1 ☐ Butter (80% fat)

_2 ☐ ”Bregott” (butter + rapeseed oli), 80% fat

_3 ☐ Margarine, 60% fat

_4 ☐ Margarine (e.g. Milda, Ädel, HushållsEve, 80% fat)

_5 ☐ Light margarine (e.g. Lätta, Becel, Lätt&Lagom, 40% fat)

_6 ☐ Light maragarine (e.g. Benecol, Leva, Becel pro.aktiv 40% fat)

_7 ☐ Olive oil

_8 ☐ Other

_9 ☐ Do not know

_10 ☐ Do not use any type of fat on bread

---

f8q6 What type of cheese does your child usually eat, and how much per week <u>or</u> per months?

	Slices/tbs per day	Slices/tbs per week	Slices/tbs per months
_1 ☐ Cheese (more than 24% fat)	a_1	b_1	c_1
_2 ☐ Cheese (less that 17% fat)	a_2	b_2	c_2
_3 ☐ Dessert cheese	a_3	b_3	c_3
_4 ☐ Spread cheese	a_4	b_4	c_4
_5 ☐ Cottage cheese	a_5	b_5	c_5
_6 ☐ Do not eat cheese			

f8q7 What type of fat does your family usually use for cooking?

(More than one option possible)

- _1 ☐ Butter
- _2 ☐ ”Bregott” (butter + rapeseed oil), 80% fat
- _3 ☐ Margarine (e.g. Milda, Ädel, HushållsEve, 80% fat)
- _4 ☐ Margarine (e.g. Flora, Linnéa)
- _5 ☐ Liquid margarine
- _6 ☐ Cooking oil
- _7 ☐ Olive oil
- _8 ☐ Other
- _9 ☐ Do not know
- _10 ☐ Do not use fat for cooking

---

f8q8 How much sugar or honey does your child usually use for tea, porridge, sour milk etc. altogether per day or week.

- a [|] table spoons per day
- b [|] table spoons per week
- c
  - 1 ☐ Eat sugar or honey less than once a month
  - 2 ☐ Never eat sugar or honey

---

f8q9 Has your child taken any dietary supplements during the last 12 months?

- 1 ☐ Yes, regularly
- 2 ☐ Yes, sometimes
- 3 ☐ No

---

f8q10 If yes, which of supplements has your child taken?

		Hur många tabl i veckan?	Sedan vilket år?
_1	☐ Multivitamins with minerals	a_1	b_1
_2	☐ Multivitamins without minerals	a_2	b_2
_3	☐ AD-vitamins	a_3	b_3
_4	☐ Vitamin A	a_4	b_4
_5	☐ Vitamin D	a_5	b_5
_6	☐ Vitamin C	a_6	b_6
_7	☐ Other, what? f8q10_7t	a_7	b_7

f8q11 How often has your child on average eaten the following food items at home and in school during the last 12 months?

FOOD ITEM		Never	Less than 1 time per month	1-3 times per month	Times per week				Times per day		
					1	2	3-4	5-6	1	2	3+
**CEREALS**		1	2	3	4	5	6	7	8	9	10
Oatmeal	_1										
Other porridge or gruel	_2										
Breakfast cereals	_3										
Spaghetti, macaroni, noodle	_4										
Pancakes	_5										
Pizza	_6										
Rice	_7										
**POTATOES, ROOT CROPS**											
Boiled potatoes	_8										
Mashed potatoes	_9										
Fried potatoes	_10										
Potatoes baked in the oven	_11										
French fries	_12										
Carrots	_13										
Turnips, beetroots	_14										
**MEAT**											
Sausage (fried or boiled)	_15										
Pork (not ground meat)	_16										
Beef or veal (not ground meat)	_17										
Ground meat (meatballs etc)	_18										
Ground meat sauce	_19										
Chicken, other poultry	_20										
Liver, kidney	_21										
Liver paté	_22										
Cold cuts (meat or sausage)	_23										
Black pudding	_24										
**FISH**											
Herring, mackerel	_25										
Salmon	_26										
Cod etc	_27										
Fish fingers	_28										
Caviar (e.g. Kalles kaviar)	_29										
Tuna fish	_30										
Shellfish (shrimps)	_31										
**EGG**											
Egg, omelette	_32										

FOOD ITEM		Never	Less than 1 time per month	1-3 times per month	Times per week				Times per day		
					1	2	3-4	5-6	1	2	3+
**VEGETABLES**		1	2	3	4	5	6	7	8	9	10
Cabbage, red cabbage	_33										
Cauliflower	_34										
Broccoli, brussels sprouts	_35										
Lettuce	_36										
Tomato	_37										
Cucumber	_38										
Spinach, collard	_39										
Onion, leak	_40										
Green peas	_41										
Pea soup	_42										
Beans, lentils, soy beans	_43										
**FRUIT, BERRIES**											
Orange, citrus fruits	_44										
Apple or pear	_45										
Banana	_46										
Other fruit	_47										
Berries (fresh or frozen)	_48										
Juice	_49										
Jam, marmalade	_50										
Stewed fruit, fruit soup	_51										
**CAKES, SWEETS**											
Buns	_52										
Danish pastry	_53										
Biscuit	_54										
Snap	_55										
Cake	_56										
Chocolate	_57										
Candy (not chocolate)	_58										
Ice cream	_59										
Chips	_60										
Popcorn	_61										
Nuts, almonds	_62										
Pea nuts	_63										
**OTHER**											
Creamy dressing	_64										
Salad dressing (oil based)	_65										
Mayonnaise	_66										
Cream, crème fraiche	_67										
**DRINKS**											
Tea	_68										
Fruit syrups	_69										
Soft drink	_70										
Other	_71										
